# *Saccharomyces cerevisiae* NRE1 and IRC24 Encode Paralogous Benzil Oxidoreductases

**DOI:** 10.17912/micropub.biology.000910

**Published:** 2023-08-03

**Authors:** Brandon Garcia, Kasandra J. Riley

**Affiliations:** 1 Rollins College, Winter Park, Florida, United States; 2 Chemistry Department and Biochemistry/Molecular Biology Program, Rollins College, Winter Park, Florida, United States

## Abstract

Irc24p is a benzil oxidoreductase encoded on chromosome IX of *Saccharomyces cerevisiae* . We identified a putative paralog, Nre1p, encoded 284 bp downstream. Both proteins are small, cytoplasmic, and are 52% identical (70% similar). PANTHER and PFAM analysis of the amino acid sequences and rigid pairwise structure alignment predicted a conserved active site and Rossmann folds in both, implicating NADH or NADPH as likely cofactors. We purified hexahistidine-tagged Irc24p and Nre1p. Both proteins catalyze the reduction of the diketone benzil with similar kinetics and a preference for NADPH. This is the first demonstration of in vitro function for Nre1p.

**Figure 1. Comparison of Nre1p and Irc24p structures and kinetics with benzil and NADH or NADPH:**
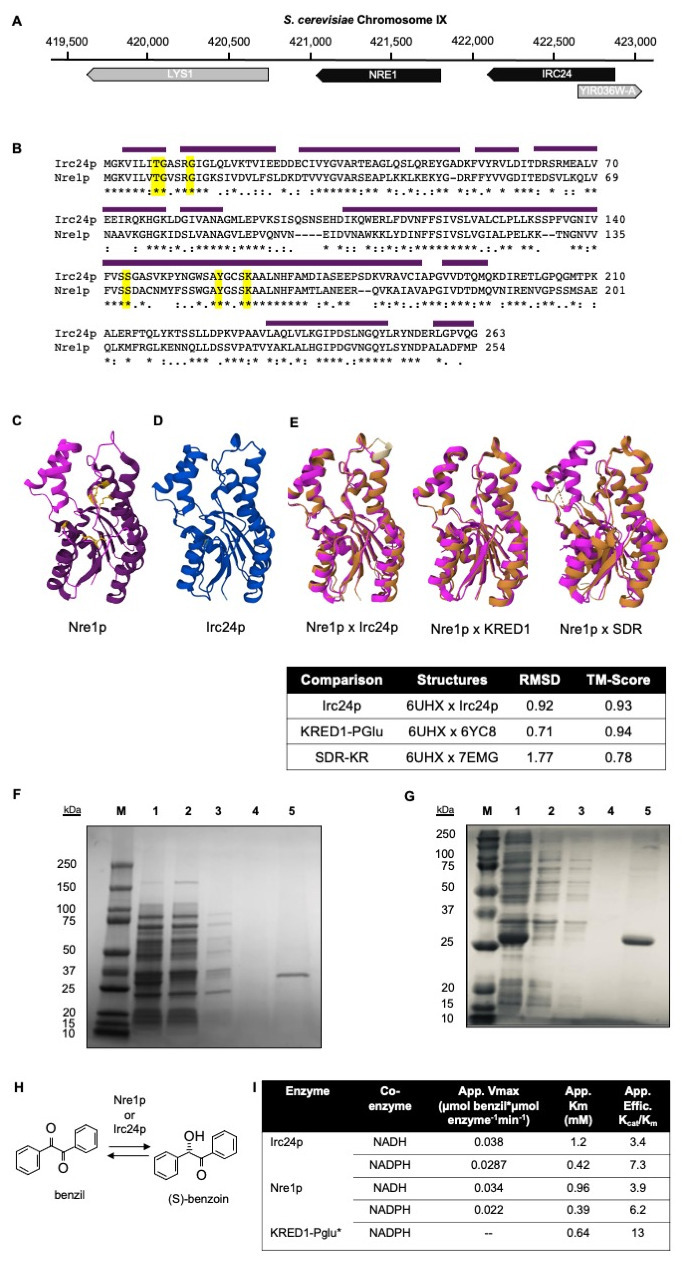
(A) Location of IRC24 (YIR036C) and NRE1 (YIR035C) on *S. cerevisiae* chromosome IX. LYS1 (YIR034C) encodes a saccharopine dehydrogenase that catalyzes the conversion of saccharopine to L-lysine. YIR036W-A is a dubious reading frame that overlaps YIR036C. (B) Clustal Omega alignment of Irc24p and Nre1p encoded by *S. cerevisiae* S288C with identity (*), high similarity (:), and low similarity (.) noted below. In yellow are the NAD(P)H binding residues S144/Y159/K164 (Irc24p) and S137/Y150/K154 (Nre1p) and the substrate binding residues T8/G9/G13 (conserved in both proteins). Amino acids in the Rossmann fold are indicated with dark purple bars above. (C) Nre1p crystal structure (deposited as PDBID 6UHX); the Rossmann fold domain is dark purple. The NAD(P)H-binding and substrate-binding residues are yellow. (D) The AlphaFold structural prediction of Irc24p. Very high confidence per-residue confidence scores (pLDDT) greater than 90/100 are depicted in royal blue. (E) Pairwise structural alignments of Nre1p/6UHX (magenta) with Irc24p/AlphaFold, oxidoreductase KRED1-Pglu/6YC8 from *P. glucozyma* , or SDR family ketoreductase (KR) from *Serratia marcescens* (PDBID: 7EMG). Irc24p, KRED1-Pglu, and SDR KR are rendered in gold *.* The pairwise alignments were calculated with original Combinatorial Extension (CE) algorithm and visualized with Mol*. The root mean square deviation (RMSD), TM-score, and percent identity of the structural alignments are reported below. (F-G) SDS-PAGE analysis of Irc24p (F) and Nre1p (G) protein purification. The lanes are as follows: M: molecular weight marker, 1: input cleared lysate, 2: unbound lysate, 3: first wash, 4: third wash, 5: imidazole eluate. Purified Irc24p and Nre1p have expected molecular weights of 31.3 kDa and 27.5 kDa, respectively. (H) Schematic of proposed redox reaction. Enantioenriched S-benzoin (2-hydroxy-1,2-diphenylethan-1-one) is the predicted product of our kinetic assays. (I) Kinetic data for Irc24p and Nre1p from *S. cerevisiae* . Literature values for KRED-Pglu from *P. glucozyma* are included for comparison (Contente, 2016). Reductase activity of Irc24p and Nre1p was measured using 0.5 mM-5 mM benzil with 0.075 mM NAD(P)H in Tris-HCl [pH 8.0] at 30˚C.

## Description

Nre1p is a 254 amino acid protein encoded by the YIR035C open reading frame (ORF) on chromosome IX of *Saccharomyces cerevisiae* ( Fig. 1A ). The Saccharomyces Genome Database (SGD) presently characterizes Nre1p as a so-called “orphan” protein, whose function is unconfirmed (Chervitz, 1999) . High-throughput protein expression data confirm that Nre1p is cytoplasmic (Kumar, 2002; Huh, 2003) .

BLASTP analysis of Nre1p indicated that a 263-amino acid protein encoded 284 bp upstream, Irc24p ( Fig. 1A ), shares significant identity (52%; Fig. 1B ). Irc24p was characterized as a stereoselective benzil reductase some 20 years ago (Maruyama, 2002) . Nre1p and Irc24p share cofactor and substrate binding residues ( Fig. 1B, yellow ) and similarity throughout. We analyzed the sequences of Nre1p and Irc24p using bioinformatic databases including PANTHER, PFAM, and the National Library of Medicine’s Conserved Domain Database (CDD), which placed both proteins in the short-chain dehydrogenases/reductases family. The CDD predicted Rossmann folds in both proteins ( Fig. 1B, dark purple ; Lu, 2020). Nre1p shares 46% amino acid identity with characterized oxidoreductase KRED1-Pglu from the non-conventional budding yeast *Ogataea glucozyma* and a 28% identity with SDR family ketoreductase from the Gram-negative bacterium *Serratia marcescens* .

The Protein Data Bank (PDB; https://www.rcsb.org/) currently reports two crystal structures (PDBID: 3KZV and 6UHX) and one computational structure (AlphaFold: AF_AFP40579F1) for Nre1p, though none of these structures is published elsewhere. Rigid pairwise structural alignment of the 100% sequence-identical Nre1p structures 3KZV and 6UHX showed a template modeling score (TM-score) of 0.84 and a root mean square deviation (RMSD) of 2.81 due to divergences limited to unstructured regions. The 6UHX structure has 253/254 modeled residues, compared to 249/254 for 3KZV, though the latter reports a better resolution (2.00 vs. 2.75 Angstroms). We selected 6UHX ( Fig. 1C ) for additional pairwise comparisons, as it aligned more closely to homologous proteins.

We used AlphaFold to predict a very high-confidence structure for Irc24p, as there is no experimentally determined structure available ( Fig. 1D ). Only five C-terminal residues (259-263) had confidence scores less than 90/100. We then conducted rigid pairwise structural alignments of Irc24p and Nre1p to determine the extent to which these proteins bear similar three-dimensional structures ( Fig. 1E ). Both the RMSD and TM-Score were close to 1, indicating a near-perfect alignment between the two structures. Additionally, we aligned Nre1p with potential homologues identified in the bioinformatic searches: KRED1-Pglu from *Pichia glucozyma* and SDR ketoreductase from *Serratia marcescens* . All structures are consistent with the short-chain dehydrogenase family, benzil reductases, and sepiapterin reductases (Mistry, 2020; Mi, 2020) . Although KRED1-Pglu and SDR-KR were each co-crystallized with NADP, structural alignments confirmed Nre1p as a structural homolog ( Fig. 1E ).

Pairwise structural alignments of the crystal structure of Nre1p and predicted structure of Irc24p also confirmed the signature Rossmann fold ( Fig. 1B and 1C, dark purple), which consists of six-stranded, extended β-sheets between a set of α-helices (Rabuffetti, 2020) . This structure is well conserved in short-chain dehydrogenase and sepiapterin reductase proteins to utilize FAD, NAD+, or NADP as a coenzyme for redox reactions with diketone compounds (Wu, 2020; Persson, 2002; Bae, 2021) . We wished to determine the preferred coenzyme and kinetics of Irc24p and Nre1p with a benzil diketone substrate. We cloned, expressed, and purified hexahisidine-tagged, full-length Nre1p and Irc24p, confirming purity using SDS-PAGE ( Fig. 1F, 1G). We tested in vitro enzyme activity with benzil and either NADH or NADPH as a coenzyme in the predicted reduction reaction ( Fig. 1H -I). Both enzymes were active with both coenzymes but showed a two-fold preference for NADPH ( Fig. 1I ). Kinetic analysis showed similar apparent Michaelis constant (Km) values for Nre1p and Irc24p and an apparent specificity constant about half of that of KRED-Pglu (Contente, 2016) . Thus, Nre1p is a benzil reductase in vitro and likely paralog of Irc24p.

## Methods

**Bioinformatic Analysis**

BLASTP was used to identify paralogous/homologous proteins within and outside *S. cerevisiae* . Amino acid sequences were aligned and depicted using CLUSTAL Omega. Irc24p and Nre1p were subjected to genome-scale phylogenetics using PANTHER (Thomas, 2021) , PFAM (Mistry, 2021) , and Conserved Domain Database (CDD; Lu, 2020) to predict protein domains and membership in protein families.

**Structure Analysis**

Crystal structures for Nre1p (3KZV and 6UHX, both unpublished), KRED1-Pglu from *P. glucozyma* (6YC8; Rabuffetti, 2021), and SDR ketoreductase from *S. marcescens* (7EMG; Wang, 2021) were retrieved from the Protein Data Bank. The structure of Irc24p was predicted using the AlphaFold Protein Structure Database. All structures were visualized and rendered in Mol* (Sehnal, 2021) . Rigid pairwise structural alignments were calculated with the original Combinatorial Extension (CE) algorithm using default settings (Shindyalov and Bourne, 1998) .

**Plasmid Construction**

The nucleotide sequences for full-length IRC24 (YIR036C; SGDID:S000001475, Chr IX from 422865-422074) and NRE1 (YIR035C; SGDID:S000001474, Chr IX from 421790-421026) were used to design *E. coli* codon-optimized GeneBlocks (IDT). The GeneBlocks were each inserted between XhoI and NheI in pET28b+ (Novagen) and verified by sequencing (Eurofins).

**Protein Expression, Purification, and Analysis**

Freshly transformed BL21(DE3)PlysS *E. coli* cells (Novagen) were grown to saturation at 37˚C with shaking (250 RPM) in LB broth with 50 µg/ml kanamycin and 34 µg/ml chloramphenicol. Cultures were diluted 1:50 in LB broth with 50 µg/ml kanamycin and induced with 1 mM isopropyl β-D-1-thiogalactopyranoside (IPTG) after reaching an OD ~0.6 (mid-log phase). Small analytical samples were collected at 2, 4-, 6-, 8-, and 24-hours post-induction for expression optimization. Samples were prepared with Laemmli buffer and beta-mercaptoethanol plus brief sonication and separated using a Mini-PROTEAN TGX 4-20% gradient SDS-PAGE gel (BioRad) stained in InstantBlue Commassie Protein Stain (Abcam). Cells from 100 ml preparative cultures collected were collected by centrifugation at 4˚C (3,000 x g for 20 min) and frozen at -20˚C until purification. Cell pellets were resuspended with 500 uL of Bacterial Protein Expression Reagent (B-PER; Thermo Scientific) with Roche EDTA-free protease inhibitors (Roche Diagnostics), lysozyme (0.2 mg/mL), and DNAaseI (1 U/uL). Lysate was incubated for 15 minutes at room temperature while mixing. The lysate was sonicated on ice three times for five seconds (setting 3) using a 550 Sonic Dismembrator (Thermo). The lysate was cleared by centrifugation at 15,000 x g for five minutes at 4 ^o^ C. The cleared supernatant was added to prepared Ni-NTA agarose beads and incubated for 45 minutes at room temperature according to the manufacturer’s instructions (Invitrogen; R90110). The beads were washed three times in wash buffer (10 mM Tris-HCl [pH 8.0], 100 mM KCl, 0.1 mM EDTA, 20 mM imidazole) and eluted twice in elution buffer (10 mM Tris-HCl [pH 8.0], 100 mM KCl, 0.1 mM EDTA, 200 mM imidazole). The eluted sample was dialyzed into 50 mM Tris-HCl, [pH 8.0] using 16 mm dry SnakeSkin 3.5K MWCO (Thermo). Sterile 100% glycerol was added 1:1 and proteins were stored briefly at -20 ^o^ C. Bradford assays quantified final yields of 9.46 uM and 9.13 uM, IRC24 and NRE1, respectively.

**Oxidoreductase Assays**

The enzyme activity assay contained purified IRC24 or NRE1 (1.2 µM), benzil (0.5 mM, 2.5 mM, 5 mM) in ddH _2_ O, 0.075 mM NAD(P)H and 10 mM Tris-HCl [pH 8.0]. The mixture was equilibrated to 30 ^o^ C for five minutes before adding substrate. Like the previously described method (Maruyama et al., 2002) , the reductase activities of IRC24 and NRE1 were spectrophotometrically assayed at 30 ^o^ C, and a decrease in absorbance was followed at 340 nm. Enzyme kinetic studies were repeated with these conditions. The enzyme kinetic constants were determined from quadruplicate samples by assuming apparent Michaelis-Menten kinetics using Lineweaver-Burk analysis.

## Reagents

*E. coli* strains:

**Table:** 

Strain	Genotype	Identifier
NEB5α	*fhuA2Δ(argF-lacZ)U169 phoA glnV44 Φ80Δ(lacZ)M15 gyrA96 recA1 relA1 endA1 thi-1 hsdR17*	Catalog# C2987I
BL21 (DE3) PLysS	*huA2 [lon] ompT gal (λ DE3) [dcm] ∆hsdSλ DE3 = λ sBamHIo ∆EcoRI-B int::(lacI::PlacUV5::T7 gene1) i21 ∆nin5*	Catalog# C257I

Reagents:

**Table:** 

Chemical	Source	Identifier
Benzil, 99.0%+	Fisher Science	Catalog B005025G
NADH disodium salt	MilliporeSigma Roche	Catalog 10128023001
NADPH tetrasodium salt	MilliporeSigma Calbiochem	Catalog GS07F161BA
pET28b(+)	Millipore Sigma Novagen	Catalog 69744

**Table:** 
